# Livestock-Associated Methicillin-Resistant *Staphylococcus aureus* in Patients Admitted to Kuwait Hospitals in 2016–2017

**DOI:** 10.3389/fmicb.2019.02912

**Published:** 2020-01-08

**Authors:** Samar S. Boswihi, Edet E. Udo, Bindu Mathew, Bobby Noronha, Tina Verghese, Sajida B. Tappa

**Affiliations:** Department of Microbiology, Faculty of Medicine, Kuwait University, Kuwait City, Kuwait

**Keywords:** livestock-associated methicillin-resistant *Staphylococcus aureus*, molecular typing, DNA microarray, antibiotic resistance genes, virulence factors

## Abstract

Livestock-associated methicillin-resistant *Staphylococcus aureus* (LA-MRSA) has been reported to colonize and cause infections in animals as well as in humans. LA-MRSA isolates have only recently been identified in patients admitted to Kuwait hospitals. This study was conducted to characterize LA-MRSA isolates obtained from patients admitted to Kuwait hospitals. A total of 202 (7.1%) of 2,823 MRSA isolates obtained from clinical samples in 2016 and 2017 in 11 public Kuwait hospitals were assigned to lineages previously known to be associated with livestock. They were characterized using antibiogram, *spa* typing, and DNA microarray for the assignment of clonal complexes (CCs) and detection of antibiotic resistance and virulence determinants. Identification as putative LA-MRSA clones was based on the molecular definition inferred from DNA microarray. The LA-MRSA isolates consisted of CC96 (*N* = 31), CC97 (*N* = 169), and CC398 (*N* = 2). Isolates belonging to CC96 and CC398 were resistant to erythromycin and clindamycin mediated by *erm*(*A*) and *erm*(C). CC97 isolates were multiresistant to gentamicin, kanamycin, erythromycin, clindamycin, tetracycline, chloramphenicol, fusidic acid, trimethoprim, and ciprofloxacin and harbored *aacA-aphD*, *erm*(A), *erm*(C), *msr*(A), *tet*(K), *cat*, *fusC*, and *dfrS1*. In total, 35 *spa* types were identified among the isolates. CC398 isolates consisted of t899 and t034. Ten *spa* types were identified among CC96 with t11822 (*N* = 13) as the most prevalent. CC97 consisted of 26 *spa* types with most belonging to t267 (*N* = 73) followed by t359 (*N* = 39). CC398 was composed of CC398-MRSA-IV and CC398-MRSA-V (PVL^+^). CC96 belonged to CC96-MRSA-IV and CC96-MRSA-IV (PVL^+^) Central Asian caMRSA/WA MRSA-119. CC97 consisted of six strains including CC97-MRSA-V (*fusC*^+^), CC97-MRSA-IV WA MRSA-54/63, CC97-MRSA-V, CC97-MRSA-(V+*fus*), CC97-MRSA-(*mec* VI+*fus*), and CC97-MRSA (*mec*V/V_T_+*fus*+*ccrAB*2). Whereas CC96 and CC97 isolates were identified in 2016 and 2017, CC398 isolates were detected only in 2016. This study identified four LA-MRSA clones among MRSA isolated from patients in Kuwait hospitals in 2016–2017 with CC97-MRSA-V (*fusC*^+^) as the dominant clone. The presence of LA-MRSA with different genetic backgrounds suggests its independent acquisition from different sources.

## Introduction

Methicillin-resistant *Staphylococcus aureus* (MRSA) is an opportunistic pathogen that colonizes humans as well as animals (Kluytmans et al., 1997). In humans, MRSA causes a wide range of infections including skin and soft tissue infections (SSTIs), and invasive infections such as pneumonia and endocarditis (Gordon and Lowy, 2008). Since it was first reported in the United Kingdom (Jevons, 1961), MRSA has evolved into three types, including health-care-associated MRSA (HA-MRSA), which was isolated from patients in health-care settings (Campanile et al., 2010); community-acquired MRSA (CA-MRSA), which was initially isolated from healthy individuals with no previous exposure to health-care facilities (Udo et al., 1993); and the new strains that were associated with livestock in the early 2000s (Armand-Lefevre et al., 2005; Voss et al., 2005), which were designated as livestock-associated MRSA (LA-MRSA). Initially, livestock-associated *S. aureus* isolates causes major problems in agriculture and are the leading cause of bovine mastitis (Fluit, 2012). In addition, MRSA isolates associated with livestock (called LA-MRSA) belonging to specific lineages such as ST398 have been reported to cause infections in animals and animal handlers (Armand-Lefevre et al., 2005; Voss et al., 2005). In recent years, these LA-MRSA lineages were able to break the species barrier to colonize and cause infections in humans with or without contact with livestock (Fitzgerald, 2012; Hetem et al., 2013).

Several molecular typing methods including staphylococcal protein A (*spa*) typing, multilocus sequence typing (MLST), pulsed-field gel electrophoresis, staphylococcal cassette chromosome *mec* (SCC*mec*) typing, DNA microarray, and whole genome sequencing (WGS) have been used to characterize and identify *S. aureus* lineages including LA-MRSA. Several LA-MRSA lineages have been identified including CC398 and CC9, which are predominant in Europe and Asia, respectively (Lo et al., 2012; Chuanga and Huang, 2015). Other clones associated with livestock include ST72, ST97, ST5, ST1, and ST433 (Lo et al., 2012; Chuanga and Huang, 2015).

The SCC*mec* genetic element is a mobile genetic element that confers methicillin resistance and resistance to other beta-lactam antibiotics to susceptible strains following its acquisition. The SCC*mec* element is variable in structural organization and the carriage of additional genetic structures such as transposons and insertion sequence elements. The high diversity in its structural organization and composition has formed the basis of SCC*mec* typing of MRSA strains (Hiramatsu et al., 2013). HA-MRSA isolates carry SCC*mec* types I, II, and III; CA-MRSA isolates carry SCC*mec* types IV, V, and VI; and LA-MRSA can carry any of the SCC*mec* types associated with CA-MRSA or HA-MRSA. For example, CC9 isolated from pigs in Asia were reported to harbor SCC*mec* III, SCC*mec* V, or SCC*mec* IX element (Cui et al., 2009; Neela et al., 2009). Similarly, CC398 isolates have been reported to carry different SCC*mec* types including SCC*mec* IV, SCC*mec* V, and SCC*mec* IX (van Loo et al., 2007).

*Staphylococcus aureus* is endowed with multiple virulence factors, such as toxins, enzymes, hemolysins, and leukocidins including Panton–Valentine leukocidin (PVL), that enhance the capacity of the bacterium to cause disease in humans. Genomic studies have revealed that LA-MRSA clones such as CC398 lack or rarely carry specific virulence factors including PVL and toxic shock syndrome toxin (TSST), which are considered major contributors in *S. aureus* infections (Jamrozy et al., 2012; Price et al., 2012). PVL is a pore-forming cytotoxin that plays a major role in *S. aureus* infections by targeting leukocytes (Maltezou and Giamarellou, 2006).

Although HA-MRSA and CA-MRSA have been widely reported in patients attending Kuwait hospitals (Boswihi et al., 2016), LA-MRSA has only recently been detected among MRSA isolates obtained from patients admitted to Kuwait hospitals. This paper reports the molecular characterization of LA-MRSA isolates obtained from patients in Kuwait hospitals in 2016–2017. LA-MRSA isolates selected for this study were identified based on their clonal complex (CC), which was determined by DNA microarray.

## Materials and Methods

### Ethical Approval

The study did not require ethical approval, because all the MRSA isolates were obtained as part of routine diagnostic microbiology investigations.

### Methicillin-Resistant *Staphylococcus aureus* Strains

A total of 4726 MRSA isolates were obtained from clinical samples in 2016 (*N* = 2305) and 2017 (*N* = 2421) in 11 public Kuwait hospitals. MRSA isolates were obtained from clinical samples submitted to the clinical microbiology diagnostic laboratory in the 11 hospitals. The isolates were identified using biochemical tests and tube coagulase at the diagnostic microbiology laboratory. Once it was identified as MRSA in the diagnostic laboratories, the isolates were sent to MRSA Reference Laboratory located in the Department of Microbiology, Faculty of Medicine, Kuwait University, for molecular typing. The information accompanying the submitted MRSA isolates was sample ID, date of isolation, patient location, patient ID, and clinical source. The isolates were subcultured twice on brain–heart infusion agar (BHIA) plates to obtain pure colonies and incubated at 35°C for 18 h. Pure cultures were preserved in beads and stored at −20 and −80°C. They were recovered on BHIA and incubated at 35°C prior to testing.

### Antibiotic Susceptibility Testing

Antibiotic susceptibility testing was performed using the disc diffusion method according to the Clinical Laboratory Standards Institute (CLSI) (Clinical and Laboratory Standard Institute, 2015). The susceptibility testing was performed with 13 antibiotics including benzyl penicillin (10 U), cefoxitin (30 μg), kanamycin (30 μg), mupirocin (200 and 5 μg), gentamicin (10 μg), erythromycin (15 μg), clindamycin (2 μg), chloramphenicol (30 μg), tetracycline (10 μg), trimethoprim (2.5 μg), fusidic acid (10 μg), rifampicin (5 μg), and ciprofloxacin (5 μg). Minimum inhibitory concentration (MIC) for cefoxitin, vancomycin, and teicoplanin were determined with Etest strips (bioMérieux, Marcy l’Etoile, France) according to the manufacturer’s instructions. *S. aureus* strains ATCC25923 and ATCC29213 were used as quality control strains for the disc diffusion and MIC determination, respectively. The *D*-test was used to test for inducible resistance to clindamycin. Susceptibility to fusidic acid was interpreted according to the British Society to Antimicrobial Chemotherapy (BSAC) (British Society to Antimicrobial Chemotherapy [BSAC], 2013).

### DNA Isolation for Amplification

Three to five identical colonies of an overnight culture were picked using a sterile loop and suspended in a microfuge tube containing 50 μl of lysostaphin (150 μg/ml) and 10 μl of RNase (10 μg/ml) solution. The tube was incubated at 37°C in the heating block (ThermoMixer, Eppendorf, Hamburg, Germany) for 20 min. To each sample, 50 μl of proteinase K (20 mg/ml) and 150 μl of Tris buffer (0.1 M) were added and mixed by pipetting. The tube was then incubated at 60°C in the water bath (VWR Scientific Co., Shellware Lab, United States) for 10 min. The tube was transferred to a heating block at 95°C for 10 min in order to inactivate proteinase K activity. Finally, the tube was centrifuged, and the extracted DNA was stored at 4°C till used for PCR.

### *Spa* Typing

Amplification of *spa* gene was performed using synthetic primers published previously (Harmsen et al., 2003) in a total volume of 25 μl. The PCR protocol consisted of an initial denaturation at 94°C for 4 min, followed by 25 cycles of denaturation at 94°C for 1 min, annealing at 56°C for 1 min, and extension for 3 min at 72°C, and a final cycle with a single extension for 5 min at 72°C. Five microliters of the PCR product was analyzed by 1.5% agarose gel electrophoresis to confirm amplification. The PCR product was purified using MicroElute Cycle-Pure Spin kit (Omega Bio-Tek Inc., United States) according to the manufacturer’s protocol. The purified DNA was used for sequencing PCR. A total of 10 μl of the sequencing reaction mixture containing 2 μl of big dye terminator mix, 2 μl of 5× sequencing buffer, 3 μl of nuclease-free water, 1 μl of 3.2 pM primer (forward and reverse), and 2 μl of purified DNA were prepared. The sequencing PCR protocol consisted of initial denaturation for 1 min at 94°C, followed by 25 cycles of denaturation for 10 s at 96°C, annealing at 55°C for 5 s, and extension for 4 min at 66°C. Ultra-Sep Dye Terminator Removal kit (Omega Bio-Tek Inc., United States) was used to purify DNA. Purified DNA was sequenced in an automated 3130 × 1 genetic analyzer (Applied Biosystems, United States) in accordance with the manufacturer’s protocol. The sequence of *spa* gene was analyzed using the Ridom Staph Type software (Ridom GmbH, Wurzburg, Germany). The software detected the *spa* repeats and assigned the *spa* type for each isolate.

### DNA Microarray

The *S. aureus* Genotyping kit 2.0 (Alere GmbH, Germany) was used for clonal assignment and the detection of genes encoding antibiotic resistance and virulence factors for MRSA isolates representing each *spa* type identified using the protocol provided by the manufacturer (Monecke et al., 2008).

## Results

### Detection of Livestock-Associated Methicillin-Resistant *Staphylococcus aureus* Isolates

A total of 4,726 MRSA isolates collected from patients in 11 hospitals in 2016–2017 were investigated by *spa* typing. From this, 2,823 isolates selected on the basis of *spa* types were analyzed by DNA microarray to assign the isolates into CCs. The 2,823 isolates included isolates from all clinical samples in all hospitals with the same *spa* types. The DNA microarray results identified 202 (7.1%) of the 2,823 isolates as LA-MRSA isolates. The isolates were defined as LA-MRSA solely on the basis of molecular rather than epidemiological definition. The LA-MRSA isolates that belonged to CC96 (31 isolates), CC97 (169 isolates), and CC398 (2 isolates) were identified as LA-MRSA. The CC96 isolates were obtained in 2016 (*N* = 21) and 2017 (*N* = 10). Eighty-three and 86 CC97 isolates were isolated in 2016 and 2017, respectively. CC398 isolates were only detected in 2016. Of the non-LA-MRSA isolates, the dominant CCs were CC5 (*N* = 796), CC22 (*N* = 397), CC8 (*N* = 304), CC1 (*N* = 239), CC6 (*N* = 223), CC30 (*N* = 179), CC80 (*N* = 178), and CC88 (*N* = 88). This report focuses on the characteristics of the LA-MRSA isolates.

The characteristics of the LA-MRSA are summarized in Table 1. The results for each isolate is presented in the Supplementary Table S1. The LA-MRSA isolates were obtained from clinical samples of patients treated in Kuwait hospitals. The samples were collected from 137 inpatients and 50 outpatients. The locations of 17 patients were not specified. The isolates were obtained from nasal swab (47; 23.2%), skin and soft tissue (47; 23.2%), groin (13; 6.4%), high vaginal swab (HVS) (13; 6.4%), blood (12; 5.9%), sputum (12; 5.9%), throat (10; 4.9%), eye (5; 2.4%), trachea (4; 1.9%), urine (4; 1.9%), axilla (2; 0.9%), ear (2; 0.9%), and fluid (1; 0.5%). The source of 30 (14.8%) isolates was unspecified.

**TABLE 1:** Genotypic characterization of LA-MRSA isolates.

**Clonal complex (CC)**	**Specimen**	**LA-MRSA strain**	**Number of isolates**	***Spa* type**
CC398	Nasal	CC398-MRSA-IV	1	t899 (1)
	Swab	CC398-MRSA-V (PVL^+^)	1	t034 (1)
CC96	Nasal (11), sputum (1), throat (2), groin (2), wound (4), skin (6), blood (1), unspecified (3)	CC96-MRSA-IV	30	t1028 (2), t11822 (13), t1198 (1), t1234 (1), t14838 (2), t203 (1), t4955 (4), t8154 (2), t8731 (1), t9867 (2), ND (1)
	Pus	CC96-MRSA-IV (PVL^+^), Central Asian caMRSA/WA MRSA-119	1	t8731 (1)
CC97	Groin	CC97-MRSA- (*mec* V/V_T_+*fus*+*ccrAB*2)	1	t2297 (1)
	Unspecified	CC97-MRSA-(*mec* VI+*fus*)	2	t359 (2)
	Wound (4), nasal (2), pus (1), urine (1), swab (1), trachea (1), unspecified	CC97-MRSA-IV, WA MRSA-54/63	11	t267 (2), t359 (5), t521 (1), t1234 (1), t693 (1), ND (1)
	Wound (1), throat (1), HVS (2), groin (2), urine (1), nasal (2), ear (2), unspecified (3)	CC97-MRSA-V	14	t1234 (5), t203 (1), t267 (4), t359 (1), t4955 (1), t2297 (1), t693 (1)
	Unspecified (17), HVS (10), fluid (1), wound (17), nasal (31), skin (5), swab (2), groin (8), pus (8), blood (11), axilla (2), throat (7), eye (5), sputum (11), trachea (3), urine (2)	CC97-MRSA-V (*fusC*^+^)	139	t1814 (2), t1965 (1), t267 (67), t2734 (1), t359 (30), t376 (2), t521 (4), t527 (1), t189 (4), t16486 (1), t2802 (1), t2297 (10), t044 (1), t15069 (1), t16606 (2), t17281 (1), t17282 (1), t17330 (1), t2770 (1), t701 (1), t9638 (1), ND (5)
	HVS	CC97-MSSA (1)	1	t16903 (1)

*ND, not determined; LA-MRSA, livestock-associated methicillin-resistant *Staphylococcus aureus*; PVL, Panton–Valentine leukocidin; HVS, high vaginal swab.*

The CC398 belonged to two genotypes including PVL-negative CC398-MRSA-IV/t899 and PVL-positive CC398-MRSA-V/t034.

Thirty CC96 isolates belonged to CC96-MRSA-IV, and one isolate belonged to CC96-MRSA-IV (PVL^+^), also known as the Central Asian caMRSA/WA MRSA-119.

Seven different subtypes were identified among the CC97 isolates. These included CC97-MRSA-V (*fusC*^+^) (139 isolates), CC97-MRSA-V (14 isolates), CC97-MRSA-IV WA MRSA-54/63 (11 isolates), CC97-MRSA-(*mec* VI+*fus*) (2 isolates), CC97-MRSA-(*mec* V/V_T_+*fus*+*ccrAB*2) (1 isolate), CC97-MRSA-(V+*fus*) (1 isolate), and CC97-MSSA (1 isolate).

The LA-MRSA isolates belonged to 35 *spa* types. CC398 isolates consisted of two *spa* types, t899 and t034. Ten *spa* types were identified among CC96 isolates including t11822 (*N* = 13), t4955 (*N* = 4), t1028 (*N* = 2), t8154 (*N* = 2), t8731 (*N* = 2), t9867 (*N* = 2), t14838 (*N* = 2), t1234 (*N* = 1), t1198 (*N* = 1), and t203 (*N* = 1). *Spa* type was not determined (ND) for one isolate.

Twenty-six *spa* types were identified among CC97 isolates, with t267 (*N* = 67), t359 (*N* = 39), and t2297 (*N* = 12) as the common *spa* types in this lineage. The other *spa* types were detected less frequently. *Spa* types t203, t1234, and t4955 were identified in both CC96 and CC97 isolates (Table 1).

### Antibiotic Susceptibility Testing and Antibiotic Resistance Genes

All LA-MRSA isolates were tested for susceptibility to antimicrobial agents. The results for the disk susceptibility testing and the genetic resistance determinants are summarized in Table 2. Antibiotic susceptibility testing showed that all LA-MRSA isolates were resistant to cefoxitin and were positive for *mecA*. A total of 187 (92.5%) isolates were resistant to penicillin mediated by *blaZ.* Sixteen CC97 isolates were phenotypically resistant to penicillin by disc diffusion method but lacked *blaZ* (Haveri et al., 2005).

**TABLE 2:** Phenotypic and genotypic characteristics of LA-MRSA isolates.

**Locus**	**CC96 (*N* = 31)**	**CC97 (*N* = 169)**	**CC398 (*N* = 2)**	**Total (*N* = 202)**
**Virulence factors**				
*Sea*	30			30
*Sec*	20			22
*Sed*		2		2
*Sel*	22			22
*sek*		2		2
*seq*		2		2
*tst*		1		1
PVL	1		1	2
PVL (P83)		4		4
*chp-scn-sak* (Type B)	30		2	32
*scn-sak* (Type E)	1	196		197
*can*	31		2	33

**Antibiogram/antibiotic resistance genes**				

**Penicillin resistance**				
Penicillin (phenotypic)	28 (90.3%)	157 (92.8%)	1 (50%)	188 (92.1%)
*blaZ*	31	141	1	175
** Methicillin-resistance**				
Cefoxitin (phenotypic)	31 (100%)	169 (100%)	2 (100%)	204 (100%)
*mecA*	31	169	2	204
** MLS-B resistance**				
Erythromycin (phenotypic)	26 (83.8%)	12 (7.1%)	2 (100%)	42 (20.5%)
Clindamycin (phenotypic)	26 (83.8%)	7 (4.1%)	2 (100%)	35 (17.1%)
*erm*(A)			1	1
*erm*(C)	26	4	1	31
*msr*(A)		3		5
Aminoglycoside resistance				
Gentamicin (phenotypic)	1 (3.2%)	120 (71.0%)		121 (59.3%)
Kanamycin (phenotypic)	1 (3.2%)	119 (70.4%)		120 (58.8%)
*aacA-aphD*		118		118
** Trimethoprim resistance**				
Trimethoprim (phenotypic)		4 (2.3%)	1 (50%)	5 (2.4%)
*dfrS1*		2	1	3
** Fusidic resistance**				
Fusidic acid (phenotypic)	2 (6.4%)	138 (81.6%)		140 (68.6%)
*fusC*		142		142
*fusB*	1	1		2
**Tetracycline resistance**				
Tetracycline (phenotypic)		30 (17.7%)	2 (100%)	32 (15.6%)
*tet*(K)		27		27
*tet*(M)			1	1
**Chloramphenicol resistance**				
Chloramphenicol (phenotypic)		1 (0.59%)		1 (0.49%)
*cat*		2		2
**Streptogramin A resistance**				
*vga*(A)		3	1	4
**Fosfomycin resistance**				
*fosB*		2		4

*LA-MRSA, livestock-associated methicillin-resistant *Staphylococcus aureus*; PVL, Panton–Valentine leukocidin.*

Gentamicin resistance and kanamycin resistance were detected in 120 CC97 isolates, with only 118 isolates positive for *aacA–aphD*. One CC96 and two CC97 isolates were phenotypically resistant to gentamicin and kanamycin but lacked any of the aminoglycoside resistance genes in the DNA microarray panel (Table 2).

Macrolide–lincosamide–streptogramin-B (MLS-B) resistance was detected in 20.7% of the LA-MRSA. All CC96 isolates resistant to erythromycin and clindamycin carried *erm*(C). MLS-B resistance in the CC398 isolates was mediated by *erm*(A) and *erm*(C). Four CC97 erythromycin- and clindamycin-resistant isolates carried *erm*(C), three isolates were resistant only to erythromycin carried *msr*(A), and five isolates phenotypically resistant to erythromycin and clindamycin lacked any of the MLS-B resistance genes in the microarray panels.

Fusidic acid resistance genes *fusB* and *fusC* were identified in CC96 and CC97 isolates. Two CC96 isolates were phenotypically resistant to fusidic acid, but only one isolate carried *fusB*. *fusB* was also detected in one isolate belonging to CC97, whereas 142 isolates carried *fusC* (Table 2).

Tetracycline resistance was detected in 30 CC97 isolates with 27 isolates carrying *tet*(K). Tetracycline resistance gene, *tet*(M), was found in one CC398 isolate (Table 2). Trimethoprim resistance gene *dfrS1* was detected in one CC398 isolate and in two of the four phenotypically resistant CC97 isolates. Chloramphenicol resistance mediated by *cat* was detected in a single CC97 isolate (Table 2).

*vga*(A), which confers resistance to streptogramin A compound (not tested phenotypically in this study), was found in three CC97 isolates and one isolate belonging to CC398. Fosfomycin was not tested in this study. However, two isolates belonging to CC97 carried *fosB*, which confers fosfomycin resistance (Table 2).

### Virulence Encoding Genes

DNA microarray demonstrated that all LA-MRSA isolates carried genes for virulence factors including genes for adhesions, accessory gene regulators (*agr*), capsular polysaccharides (*cap*), and enzymes but varied in their carriage of genes for exotoxins (Table 2). All isolates were positive for *agr* and *cap* but differed in the types of *agr* and *cap* alleles. CC398 and CC97 carried *agr* 1 and *cap* 5, whereas CC96 isolates carried *agr* III and *cap* 8.

Of the 204 LA-MRSA isolates, 80 (39.6%) isolates carried enterotoxins. Two CC97 isolates carried *sed*, *sek*, and *seq*, whereas CC96 isolates variably carried *sec*, *sea*, and *sel* (Table 2). No enterotoxins were detected among CC398 isolates (Table 2).

Gene for TSST-1, *tst*, was detected in one CC97 isolate. PVL was only found in two CC96 isolates.

All isolates carried microbial surface components recognizing adhesive matrix molecules (MSCRAMMs). However, they varied in the carriage of collagen adhesion (*cna*). The *cna* gene was detected in CC96 and CC398 isolates but not in CC97 isolates (Table 2).

The immune evasion cluster (IEC) genes (*scn*, *chp*, and *sak*) were identified in all LA-MRSA isolates. CC398 carried *scn*, *chp*, and *sak* encoding genes (IEC type B). All CC97 and one CC96 isolates lacked *chp* and carried *scn* and *sak* (IEC type E).

## Discussion

Livestock-associated methicillin-resistant *S. aureus* was initially isolated from livestock and later in isolates from humans who were in contact with livestock (Kinross et al., 2017). Subsequently, LA-MRSA isolated from individuals with no contact with livestock was reported in different places including Italy (Pan et al., 2009), Spain (Lozano et al., 2011), Australia (Monecke et al., 2011), and Saudi Arabia (Monecke et al., 2012). In this study, we characterized LA-MRSA obtained from human patients in Kuwait hospitals. The result of the study revealed that LA-MRSA constituted 7.1% of MRSA isolated from patients in hospitals in Kuwait in 2016–2017. The low prevalence of LA-MRSA reported in this study is similar to results reported in patients in Europe including the United Kingdom (Harrison et al., 2017); Luxembourg, Poland, and Norway (Kinross et al., 2017); and Asian countries such as China, Taiwan, Japan, and Malaysia (Chuanga and Huang, 2015). Human colonization with LA-MRSA is more common in areas with high density of livestock (Cuny et al., 2013; Kinross et al., 2017). Nevertheless, colonization with LA-MRSA isolates was also reported in people with no contact with livestock. A study published in 2013 in the United States reported CC398 MSSA as the dominant strain among detainees in the Dallas County Jail (David et al., 2013), which showed the transmission of these isolates in the absence of an animal source.

The LA-MRSA isolates belonged to three CCs, including CC96, CC97, and CC398, in this study. CC97 was found in 169 isolates, making CC97 the dominant LA-MRSA clone in Kuwait hospitals in 2016–2017. Prior to this report, CC97 isolates were reported to cause an outbreak in a neonatal intensive care unit (ICU) in a Kuwait hospital in 2007 (Udo et al., 2011; Udo and Al-Sweih, 2017) and was detected in four isolates in another hospital in 2010 (Boswihi et al., 2016). The increase in the prevalence of CC97 in recent years in Kuwait may suggest an increased transmission among patients in hospitals. CC97 has also been sporadically reported in patients in Saudi Arabia (Monecke et al., 2012), Spain (Lozano et al., 2011; Reynaga et al., 2018), and Australia (Monecke et al., 2011). Although CC97 is rarely reported in humans (Grundmann et al., 2010; Schaumburg et al., 2012), it is commonly isolated from cattle, and it is considered one of the most common causes of bovine mastitis (Smith et al., 2005; Aires-de-Sousa et al., 2007; Sung et al., 2008) and one of the most common in the Italian pig industry (Battisti et al., 2010; Feltrin et al., 2016). The increase in the prevalence of CC97 among human patients observed in this study is of concern, as it highlights the increasing transmission of LA-MRSA among human patients. The CC97 isolates consisted of 26 *spa* types and six genotypes, revealing the diversity of the isolates. CC97-MRSA-V (fusC^+^)-t267 was the dominant strain carrying few enterotoxin genes (*sed*, *sek*, and *seq*) and bovine PVL (P83), which is similar to the bovine CC97 isolates reported in Italy (Feltrin et al., 2016). *Spa* type t267 was also reported in isolates obtained from bovine in Portugal (Conceição et al., 2017), Switzerland (Boss et al., 2016), and Brazil (Rabello et al., 2007). The other *spa* types associated with CC97 isolates in this study (t359, t521, t2297) have also been reported in patients as well as animals in other studies (Albrecht et al., 2015; Feltrin et al., 2016), making them successful zoonotic subtypes.

Few reports have described the virulence profiles of CC97 isolates. A study in South Africa (Schmidt et al., 2017) showed that CC97 isolated from bovine and humans carried few enterotoxins genes including *sec* and *sel*. Similarly, the CC97 isolates in this study harbored *sec* and *sel*, suggesting that *sec* and *sel* may be common features of CC97 MRSA isolates.

CC97 isolates in this study were multiresistant to antibiotics, including resistance to gentamicin, kanamycin, erythromycin, clindamycin, and tetracycline. Similarly, multiresistant strains of CC97 were isolated from human patients in Saudi Arabia (Monecke et al., 2012) and in bovine in Spain (Gómez-Sanz et al., 2010). In contrast, non-multiresistant isolates of CC97 were obtained from dairy milk in China (Wang et al., 2018). We observed differences between the antibiotic susceptibility patterns and the carriage of antibiotic resistance genes in CC97 isolates obtained in this study. Penicillin resistance was detected in 157 of the CC97 isolates, but only 141 carried *blaZ*, which could be due to a lack of signal to *blaZ*, *blaI*, and *blaR*. A similar observation was reported by Williamson et al. (2014) in two CC398-t034 isolates in which *blaZ* could not be detected by DNA microarray, although they were resistant to penicillin and the resistance was confirmed by the detection of penicillinase with nitrocefin. A similar pattern was also observed in isolates resistant to erythromycin, gentamicin, kanamycin, trimethoprim, and tetracycline, in which the corresponding resistance genes could not be detected by the arrays. This could be due to the presence of other resistance mechanisms that are not in the DNA microarray panel or due to intrinsic resistance in these isolates. Intrinsic resistance was documented in *S. aureus* with penicillin-binding protein 2a (PBP2a), which renders the effectiveness of the beta-lactam antibiotics.

CC96 was the second most common LA-MRSA clone detected in this study. Isolates from this lineage were first detected in Kuwait in 2016 (21 isolates) and then in 2017 (10 isolates). CC96 MRSA isolates are rare in humans with only single isolates reported previously from Russia (Mendes et al., 2012) and Saudi Arabia (Senok et al., 2016; Mat Azis et al., 2017). However, ST96-MSSA is a common pathogen of rabbits, where it causes different infections (Mendes et al., 2012; Viana et al., 2015; Merz et al., 2016; Moreno-Grúa et al., 2018). An isolate of ST96-MRSA belonging to *spa* type t1190 was isolated from a rabbit meat sample that could not be characterized as either CA-MRSA or HA-MRSA (European Food Safety Authority, 2017), suggesting that a previously ST96-MSSA/t1190 had acquired the *mecA* determinant. Although the ST96-MRSA in this study belongs to different spa types, we argue that they are probably related to the rabbit ST96 lineage. Furthermore, the CC96-MRSA in this study belonged to agr III and cap8 and were resistant to erythromycin mediated by *ermC* similar to ST96-MSSA isolates isolated from rabbits (Merz et al., 2016). The lack of information on the epidemiology and the genetic characteristics of ST96-MRSA in the literature warrants further studies to describe the origin, prevalence, and molecular characteristics of the emerging CC96-MRSA.

Since its discovery in the Netherlands in the early 2000s, CC398 has become the most common LA-MRSA clone circulating in Europe (Butaye et al., 2016). This is the first report of CC398 in human patients in Kuwait hospitals and as far as we know in the Gulf Cooperation Council (GCC) countries. CC398 is the most prevalent lineage in pigs (Butaye et al., 2016; Feltrin et al., 2016), but it has also been reported in horses, poultry, cattle, and companion animals (Butaye et al., 2016). The CC398-MRSA was classified into two strains carrying SCC*mec* IV and V each and belonged to *spa* types t899 and t034, respectively. The CC398-IV-t899 isolate was PVL negative, similar to isolates obtained from animals in United Kingdom (Bortolami et al., 2017) and human patients in Spain (Lozano et al., 2012), Italy (Pan et al., 2009), and Denmark (Larsen et al., 2016). MRSA belonging to spa type t899 has been described as a hybrid LA-MRSA of CC9 and CC398 (Larsen et al., 2016; Sharma et al., 2018).

Apparently, CC398-MRSA of animal origin usually lacks enterotoxins and PVL (Feltrin et al., 2016), whereas the early-branching East Asian strain is positive for PVL (Yu et al., 2008). In this study, one CC398-MRSA-V isolate was positive for PVL, as has also been reported in human patients in China (Yu et al., 2008), Sweden (Welinder-Olsson et al., 2008), Finland (Salmenlinna et al., 2010), and New Zealand (Williamson et al., 2014), suggesting that our strain may belong to the human branch of CC398. The lack of enterotoxins in these two CC398-MRSA isolates is consistent with previous results suggesting that absence of enterotoxins may be a characteristic feature of isolates from this lineage (Lozano et al., 2012; Feltrin et al., 2016).

CC398 strains of animal origin usually lack the IEC genes that facilitate the colonization and invasion of MRSA in human hosts (Price et al., 2012). The two CC398 isolates detected in this study carried the IEC genes *scn*, *chp*, and *sak* (IEC type B). Similarly, recent studies by Cuny et al. (2015) and Pérez-Moreno et al. (2017) also reported CC398 isolates of human origin carrying the IEC genes. The presence of IEC genes in these isolates may explain the ability of these strains to jump from livestock and successfully adapt and colonize human beings. One of the CC398 isolates belonged to *spa* type t899.

The major limitation of this study is the lack of information on the patients’ travel history or contact with animals. Therefore, it is difficult to determine if these LA-MRSA strains were acquired by contact with livestock or by household members who are in contact with livestock. Nevertheless, the detection of these clones in human patients is significant because it shows their expansion beyond the usual livestock hosts, which may pose new problems for infection control.

## Conclusion

In conclusion, this study described the characteristics of LA-MRSA strain belonging to CC96, CC97, and CC398 in patients in Kuwait hospitals. Genotyping showed that our isolates are diverse and belonged to different lineages including CC398, which are prevalent in Europe. This is the first report of CC398 LA-MRSA in Kuwait. The study also revealed that most of the isolates belonging to CC97 expressed resistance to multiple classes of antibiotics. The CC97 and CC398 MRSA isolates shared characteristics similar to those obtained from bovine and human patients in Europe. These observations suggest that LA-MRSA isolates were introduced to Kuwait via different routes. Further surveillance studies are required to monitor future transmission patterns of these isolates. Although LA-MRSA may have the same virulence potential and causes similar infections as *S. aureus*, identifying these isolates can inform on their origin, which will help in controlling the spread of MRSA in the clinical settings.

## Data Availability Statement

All datasets generated for this study are included in the article/Supplementary Material.

## Author Contributions

SB, BM, BN, TV, and ST carried out the laboratory work. SB performed the data analysis. EU performed the experimental design. SB and EU carried out the manuscript writing and editing. All authors read and approved the final manuscript.

## Conflict of Interest

The authors declare that the research was conducted in the absence of any commercial or financial relationships that could be construed as a potential conflict of interest. The reviewer SM declared a past co-authorship, with several of the authors, SB and EU, to the handling editor.
