# Supplementary material for: Livestock-Associated Methicillin-Resistant Staphylococcus aureus in Patients Admitted to Kuwait Hospitals in 2016–2017
Source: Front Microbiol. 2020 Jan 8;10:2912. doi: 10.3389/fmicb.2019.02912 (PMC6960094; doi:10.3389/fmicb.2019.02912)
Supplement: Supplementary file 1 [file Table_1.pdf]

Supplementary Table S1

Sample No.	CLONE	SCC MEC	Spa Type	PVL	Date	Specimen	Penicillin	Gentamicin	Kanamycin	Erythromycin	Clindamycin	Chloramphenicol	Tetracycline	Trimethoprim	Rifampicin	Fusidic Acid	Ciprofloxacin	Cefoxitin	Mupirocin 200	Vancomycin mg\L	Teicoplanin mg\L	Antibiotic resistance genes	Toxins	Miscellaneous genes
5381	**CC398-MRSA-IV**	IV	t899	-	18.05.16	Nasal	R	S	S	R	C	S	R	R	S	S	S	R	S	0.8	0.8	*blaZ, erm(C), vga(A) (BM 3327), dfrS1, tet(M), sdrM*	hld, hla, hl, hlb, hIII	agrI; cap5; sak/chp/scn; icaA/C/D
17562	**CC398-MRSA-V [PVL+]**	V	t034	+	23.08.16	Swab	S	S	S	R	C	S	R	S	S	S	S	R	S	2	3	*blaZ, erm(C), tet(M), sdrM*	PVL, hld, hla, hl, hIII	agrI; cap5; sak/chp/scn; icaA/C/D
18961	***CC96-MRSA-IV***	IV	t9867	-	28.02.17	Blood	R	S	S	R	I	S	S	S	S	S	R	R	S	1.5	1.5	*blaZ, erm(C), sdrM*	sea, sec, sel,; hld, hl, hla, hlb, hIII	agrIII; cap8; sak/chp/scn; icaA/C/D
4335	***CC96-MRSA-IV***	IV	t11822	-	03.02.16	Groin	S	S	S	R	I	S	S	S	S	S	S	R	S	1	1	*blaZ, erm(C), sdrM*	sea; hld, hl, hla, hlb, hIII	agrIII; cap8; sak/chp/scn; icaA/C/D
19204	***CC96-MRSA-IV***	IV	t8154	-	23.03.17	Groin	R	S	S	S	S	S	S	S	S	S	R	R	S	2	3	*blaZ, sdrM*	sel; hld, hl, hla, hlb, hIII	agrIII; cap8; sak/chp/scn; icaA/C/D
5385	***CC96-MRSA-IV***	IV	t11822	-	18.05.16	Nasal	R	S	S	S	S	S	S	S	S	S	R	R	S	1	1	*blaZ, sdrM*	sea, sec, sel; hld, hl, hla, hlb, hIII	agrIII; cap8; sak/chp/scn; icaA/C/D
5863	***CC96-MRSA-IV***	IV	t4955	-	17.07.16	Nasal	R	S	S	S	S	S	S	S	S	S	R	R	S	1.5	1.5	*blaZ, sdrM*	sea, sec, sel; hld, hl, hla, hlb, hIII	agrIII; cap8; sak/chp/scn; icaA/C/D
5083	***CC96-MRSA-IV***	IV	t1234	-	19.04.16	Nasal	S	S	S	R	I	S	S	S	S	S	R	R	S	1.5	1.5	*blaZ, erm(C), sdrM*	sea, sec, sel; hld, hl, hla, hlb, hIII	agrIII; cap8; sak/chp/scn; icaA/C/D
4617	***CC96-MRSA-IV***	IV	t11822	-	2.3.2016	Nasal	R	S	S	R	I	S	S	S	S	S	R	R	S	0.8	2	*blaZ, erm(C), far1, sdrM*	sea; hld, hl, hla, hlb, hIII	agrIII; cap8; sak/chp/scn; icaA/C/D
17777	**CC96-MRSA-IV**	IV	t11822	-	22.09.16	Nasal	R	S	S	R	I	S	S	S	S	S	R	R	S	1	1	*blaZ, erm(C), sdrM*	sea; hld, hl, hla, hlb, hIII	agrIII; cap8; sak/chp/scn; icaA/C/D
17332	**CC96-MRSA-IV**	IV	t4955	-	26.1.16	Nasal	S	S	S	R	I	S	S	S	S	S	R	R	S	1	1	*blaZ, erm(C), sdrM*	sea, sec, sel; hld, hl, hla, hlb, hIII	agrIII; cap8; sak/chp/scn; icaA/C/D
19046	***CC96-MRSA-IV***	IV	t11822	-	07.03.17	Nasal	R	S	S	R	I	S	S	S	S	S	R	R	S	1.5	2	*blaZ, erm(C), sdrM*	sea; hld, hl, hla, hlb, hIII	agrIII; cap8; sak/chp/scn; icaA/C/D
19490	***CC96-MRSA-IV***	IV	t11822	-	02.05.17	Nasal	R	S	S	R	I	S	S	S	S	S	R	R	S	2	2	*blaZ, erm(C), sdrM*	sea; hld, hl, hla, hlb, hIII	agrIII; cap8; sak/chp/scn; icaA/C/D
19265	***CC96-MRSA-IV***	IV	t11822	-	02.04.17	Nasal	R	S	S	R	I	S	S	S	S	R	R	R	S	1.5	2	*blaZ, erm(C), sdrM*	sea; hld, hl, hla, hlb, hIII	agrIII; cap8; sak/chp/scn; icaA/C/D
18852	***CC96-MRSA-IV***	IV	t203	-	08.02.17	Nasal	R	S	S	R	I	S	S	S	S	S	R	R	S	1.5	1.5	*blaZ, erm(C), sdrM*	sea, sec, sel; hld, hl, hla, hlb, hIII	agrIII; cap8; sak/chp/scn; icaA/C/D
20392	***CC96-MRSA-IV***	IV	ND	-	12.09.17	Nasal	R	S	S	R	I	S	S	S	S	S	R	R	S	1.5	2	*blaZ, erm(C), sdrM*	sea, sec, sel; hld, hl, hla, hlb, hIII	agrIII; cap8; sak/chp/scn; icaA/C/D
5233	***CC96-MRSA-IV***	IV	t1028	-	02.05.16	Skin	R	S	S	R	I	S	S	S	S	S	R	R	S	2	2	*erm(C), sdrM*	sec, sel; hld, hl, hla, hlb, hIII	agrIII; cap8; icaA/C/D
18004	**CC96-MRSA-IV**	IV	t11822	-	25.10.16	Skin	R	S	S	R	I	S	S	S	S	S	R	R	S	2	2	*erm(C), sdrM*	sea; hld, hl, hla, hlb, hIII	agrIII; cap8; sak/chp/scn; icaA/C/D
18205	**CC96-MRSA-IV**	IV	t11822	-	17.11.16	Skin	R	S	S	R	I	S	S	S	S	S	R	R	S	1	1.5	*erm(C), sdrM*	sea; hld, hl, hla, hlb, hIII	agrIII; cap8; sak/chp/scn; icaA/C/D
18416	**CC96-MRSA-IV**	IV	t11822	-	12.12.16	Skin	R	S	S	R	I	S	S	S	S	S	S	R	S	1.5	1.5	*erm(C), sdrM*	sea; hld, hl, hla, hlb, hIII	agrIII; cap8; sak/chp/scn; icaA/C/D
18983	***CC96-MRSA-IV***	IV	t1198	-	02.03.17	Skin	R	S	S	R	I	S	S	S	S	S	R	R	S	1	1.5	*blaZ, erm(C), sdrM*	sea, sec, sel; hld, hl, hla, hlb, hIII	agrIII; cap8; sak/chp/scn; icaA/C/D
20486	***CC96-MRSA-IV***	IV	t14838	-	26.09.17	Skin	R	S	S	R	C	S	S	S	S	S	R	R	S	1.5	1.5	*blaZ, erm(C), sdrM*	sea, sec, sel; hld, hl, hla, hlb, hIII	agrIII; cap8; icaA/C/D
5519	***CC96-MRSA-IV***	IV	t8731	-	31.05.16	Sputum	R	S	S	R	I	S	S	S	S	S	R	R	S	1	2	*blaZ, erm(C), sdrM*	sea, sec, sel; hld, hl, hla, hlb, hIII	agrIII; cap8; sak/chp/scn; icaA/C/D
4994	***CC96-MRSA-IV***	IV	t1028	-	12.04.16	Throat	R	S	S	R	I	S	S	S	S	S	R	R	S	2	1.5	*blaZ, erm(C), sdrM*	sec, sel; hld, hl, hla, hlb, hIII	agrIII; cap8; icaA/C/D
17597	**CC96-MRSA-IV**	IV	t11822	-	25.08.16	Throat	R	S	S	R	I	S	S	S	S	S	R	R	S	1.5	1	*blaZ, erm(C), sdrM*	sea, sec, sel; hld, hl, hla, hlb, hIII	agrIII; cap8; sak/chp/scn; icaA/C/D
5834	***CC96-MRSA-IV***	IV	t4955	-	12.07.16	Unknown	R	S	S	R	I	S	S	S	S	S	R	R	S	1	1	*blaZ, erm(C), sdrM*	sea, sec, sel; hld, hl, hla, hlb, hIII	agrIII; cap8; sak/chp/scn; icaA/C/D
5596	***CC96-MRSA-IV***	IV	t8154	-	07.06.16	Unknown	R	S	S	R	I	S	S	S	S	S	R	R	S	1	1.5	*blaZ, erm(C), sdrM*	sea, sec, sel; hld, hl, hla, hlb, hIII	agrIII; cap8; sak/chp/scn; icaA/C/D
5210	***CC96-MRSA-IV***	IV	t11822	-	01.05.16	Unknown	R	R	R	R	I	S	S	S	S	S	R	R	S	2	1.5	*blaZ, erm(C), sdrM*	sea, sec, sel; hld, hl, hla, hlb, hIII	agrIII; cap8; sak/chp/scn; icaA/C/D
5497	***CC96-MRSA-IV***	IV	t11822	-	29.05.16	Wound	R	S	S	R	I	S	S	S	S	S	R	R	S	1	1.5	*blaZ, erm(C), sdrM*	sea, sec, sel; hld, hl, hla, hlb, hIII	agrIII; cap8; sak/chp/scn; icaA/C/D
17949	**CC96-MRSA-IV**	IV	t9867	-	19.10.16	Wound	R	S	S	S	S	S	S	S	S	R	R	R	S	1.5	2	*blaZ, sdrM*	sea, sec, sel,; hld, hl, hla, hlb, hIII	agrIII; cap8; sak/chp/scn; icaA/C/D
18486	**CC96-MRSA-IV**	IV	t14838	-	22.12.16	Wound	R	S	S	R	I	S	S	S	S	S	S	R	S	1.5	2	*blaZ, erm(C), sdrM*	sea, sec, sel; hld, hl, hla, hlb, hIII	agrIII; cap8; sak/chp/scn; icaA/C/D
19368	***CC96-MRSA-IV***	IV	t4955	-	16.04.17	Wound	R	S	S	S	S	S	S	S	S	S	R	R	S	1.5	2	*blaZ, sdrM*	sea, sec, sel; hld, hl, hla, hlb, hIII	agrIII; cap8; sak/chp/scn; icaA/C/D
17844	**CC96-MRSA-IV [PVL+], Central Asian caMRSA/WA MRSA-119**	IV	t8731	+	06.10.16	Pus	R	S	S	R	I	S	S	S	S	S	R	R	S	1.5	1.5	*blaZ, erm(C), sdrM*	PVL, sea, sec, sel; hld, hl, hla, hlb, hIII	agrIII; cap8; sak/chp/scn; icaA/C/D
17914	**CC97-MRSA-[mec V/VT+fus+ccrAB2]**	V	t2297	-	16.10.16	Groin	R	S	S	S	S	S	S	S	S	R	S	R	S	1.5	2	*blaZ, fusC, sdrM*	hld, hl, hla, hlb, hIII	agrI; cap5; sak/scn; icaA/C/D
18335	**CC97-MRSA-[mec VI+fus]**	VI	t359	-	04.12.16	Unknown	S	S	S	S	S	S	S	R	S	R	S	R	S	2	2	*fusC, dfrS1, sdrM*	hld, hl, hla, hlb, hIII	agrI; cap5; sak/scn; icaA/C/D
20612	**CC97-MRSA-[mec VI+fus]**	VI	t359	-	16.10.17	Unknown	R	S	S	S	S	S	S	R	S	R	S	R	S	2	2	*fusC, dfrS1, sdrM*	hld, hl, hla, hlb, hIII	agrI; cap5; sak/scn; icaA/C/D
5599	***CC97-MRSA-[V+fus]***	V	t359	-	05.06.16	Unknown	R	R	R	S	S	S	S	S	S	R	S	R	S	1	1	*blaZ, aacA-aphD, fusC, sdrM*	hld, hl, hla, hlb, hIII	agrI; cap5; sak/scn; icaA/C/D
18421	**CC97-MRSA-IV, WA MRSA-54/63**	IV	t359	-	15.12.16	Nasal	R	S	S	S	S	S	S	R	S	S	S	R	S	1.5	1.5	*blaZ, sdrM*	hld, hla, hlb	agrI; cap5; sak; icaA/C/D
18240	**CC97-MRSA-IV, WA MRSA-54/63**	IV	t521	-	23.11.16	Nasal	R	S	S	R	S	S	S	S	S	S	S	R	S	1.5	1.5	*blaZ, msr(A), sdrM*	hld, hl, hla, hlb, hIII	agrI; cap5; sak/scn; icaA/C/D
18944	**CC97-MRSA-IV, WA MRSA-54/63**	IV	t359	-	27.02.17	Pus	R	S	S	S	S	S	S	S	S	S	S	R	S	1.5	2	*blaZ, sdrM*	hld, hl, hla, hlb, hIII	agrI; cap5; sak/scn; icaA/C/D
20879	**CC97-MRSA-IV, WA MRSA-54/63**	IV	ND	-	29.11.17	Swab	R	S	S	R	S	S	S	S	S	S	S	R	S	1.5	1.5	*blaZ, msr(A), sdrM*	hld, hl, hla, hlb, hIII	agrI; cap5; sak/scn; icaA/C/D
18561	**CC97-MRSA-IV, WA MRSA-54/63**	IV	t1234	-	03.01.17	Tracheal	R	S	S	S	S	S	S	S	S	S	S	R	S	1.5	2	*blaZ, sdrM*	hld, hl, hla, hlb, hIII	agrI; cap5; sak/scn; icaA/C/D
18609	**CC97-MRSA-IV, WA MRSA-54/63**	IV	t267	-	08.01.17	Unknown	R	S	S	S	S	S	S	S	S	S	R	R	S	2	2	*blaZ, sdrM*	hld, hl, hla, hlb, hIII	agrI; cap5; sak/scn; icaA/C/D
20501	**CC97-MRSA-IV, WA MRSA-54/63**	IV	t359	-	28.09.17	Urine	R	S	S	S	S	S	S	S	S	S	R	R	S	1.5	2	*blaZ, sdrM*	hld, hl, hla, hlb, hIII	agrI; cap5; sak/scn; icaA/C/D
5884	**CC97-MRSA-IV, WA MRSA-54/63**	IV	t267	-	19.07.16	Wound	R	S	S	S	S	S	R	S	S	S	S	R	S	1	1.5	*blaZ, tet(K), sdrM*	hld, hl, hla, hlb, hIII	agrI; cap5; sak/scn; icaA/C/D
20461	**CC97-MRSA-IV, WA MRSA-54/63**	IV	t359	-	20.09.17	Wound	R	S	S	S	S	S	S	S	S	S	S	R	S	1.5	2	*blaZ, sdrM*	hld, hl, hla, hlb, hIII	agrI; cap5; sak/scn; icaA/C/D
17825	**CC97-MRSA-IV, WA MRSA-54/63**	IV	t359	-	04.10.16	Wound	R	S	S	S	S	S	S	S	S	S	S	R	S	2	2	*blaZ, sdrM*	hld, hl, hla, hlb, hIII	agrI; cap5; sak/scn; icaA/C/D
19384	**CC97-MRSA-IV, WA MRSA-54/63**	IV	t693	-	12.04.17	Wound	R	S	S	S	S	S	S	R	S	S	S	R	S	2	2	*blaZ, sdrM*	hld, hl, hla, hlb, hIII	agrI; cap5; sak/scn; icaA/C/D
18559	**CC97-MRSA-V**	V	t267	-	05.01.17	Ear	R	R	R	S	S	S	S	S	S	S	S	R	S	1.5	1.5	*blaZ, aacA-aphD, sdrM*	hld, hl, hla, hlb, hIII	agrI; cap5; sak/scn; icaA/C/D
20503	**CC97-MRSA-V**	V	t693	-	28.09.17	Ear	R	S	S	S	S	S	S	S	S	S	S	R	S	2	3	*blaZ, sdrM*	hld, hl, hla, hlb, hIII	agrI; cap5; sak/scn; icaA/C/D
20245	**CC97-MRSA-V**	V	t1234	-	20.08.17	Groin	R	S	S	S	S	S	S	S	S	S	S	R	S	2	1.5	*sdrM*	hld, hl, hla, hlb, hIII	agrI; cap5; icaA/C/D
17538	**CC97-MRSA-V**	V	t267	-	21.08.16	Groin	R	R	R	S	S	S	S	S	S	S	S	R	S	2	2	*blaZ, sdrM*	hld, hlb, hIII	agrI; cap5; sak; icaA/C/D
17886	**CC97-MRSA-V**	V	t1234	-	10.10.16	HVS	R	S	S	S	S	S	S	S	S	S	S	R	S	1.5	2	*sdrM*	hld, hl, hla, hlb, hIII	agrI; cap5; sak/scn; icaA/C/D
18144	**CC97-MRSA-V**	V	t359	-	10.11.16	HVS	R	R	R	S	S	S	S	S	S	S	S	R	S	1	1.5	*blaZ, aacA-aphD, sdrM*	hld, hl, hla, hlb, hIII	agrI; cap5; sak/scn; icaA/C/D
18478	**CC97-MRSA-V**	V	t267	-	28.12.16	Nasal	R	R	R	S	S	S	R	S	S	S	S	R	S	1.5	1.5	*blaZ, aacA-aphD, tet(K), sdrM*	hld, hl, hla, hlb, hIII	agrI; cap5; sak/scn; icaA/C/D
18113	**CC97-MRSA-V**	V	t4955	-	08.11.16	Nasal	R	S	S	S	S	S	S	S	S	S	S	R	S	2	2	*blaZ, sdrM*	hld, hl, hla, hlb, hIII	agrI; cap5; sak/scn; icaA/C/D

4791	**CC97-MRSA-V**	V	t1234	-	22.3.2016	Others	R	S	S	R	I	S	R	S	S	S	S	R	S	0.8	1	*blaZ, erm(A), erm(C), tet(K), sdrM*	hld, hl, hla, hlb, hIII	agrI; cap5; sak/scn; icaA/C/D
17567	**CC97-MRSA-V**	V	t267	-	25.08.16	Others	R	R	R	S	S	S	S	S	S	S	S	R	S	0.8	1.5	*blaZ, aacA-aphD, sdrM*	hld, hl, hla, hlb, hIII	agrI; cap5; sak/scn; icaA/C/D
5944	**CC97-MRSA-V**	V	t1234	-	27.07.16	Throat	R	S	S	S	S	S	S	S	S	S	S	R	S	1	1	*blaZ, tet(K), sdrM*	hld, hl, hla, hlb, hIII	agrI; cap5; sak/scn; icaA/C/D
19212	**CC97-MRSA-V**	V	t2297	-	27.03.17	Unknown	S	S	S	S	S	S	S	S	S	R	S	R	S	2	2	*sdrM*	hld, hl, hla, hlb, hIII	agrI; cap5; sak/scn; icaA/C/D
5168	**CC97-MRSA-V**	V	t203	-	28.04.16	Urine	R	S	S	R	C	S	S	S	S	S	S	R	S	1	1.5	*blaZ, erm(C), sdrM*	hld, hl, hla, hlb, hIII	agrI; cap5; icaA/C/D
4546	**CC97-MRSA-V**	V	t1234	-	24.2.16	Wound	R	S	S	S	S	S	S	S	S	S	S	R	S	2	1.5	*tet(K), sdrM*	hld, hl, hla, hlb, hIII	agrI; cap5; sak/scn; icaA/C/D
19308	**CC97-MRSA-V [fusC+]**	V	t2297	-	06.04.17	Axilla	R	S	S	S	S	S	S	S	S	R	S	R	S	2	2	*blaZ, fusC, sdrM*	hld, hl, hla, hlb, hIII	agrI; cap5; sak/scn; icaA/C/D
17549	**CC97-MRSA-V [fusC+]**	V	t267	-	22.08.16	Axilla	R	R	R	S	S	S	S	S	S	R	R	R	S	1	2	*blaZ, aacA-aphD, fusC, sdrM*	hld, hl, hla, hlb, hIII	agrI; cap5; sak/scn; icaA/C/D
17312	**CC97-MRSA-V [fusC+]**	V	t2297	-	20.1.16	Blood	R	S	S	S	S	S	S	S	S	R	S	R	S	1	1.5	*blaZ, fusC, sdrM*	hld, hl, hla, hlb, hIII	agrI; cap5; sak/scn; icaA/C/D
4403	**CC97-MRSA-V [fusC+]**	V	t267	-	14.02.16	Blood	R	R	R	S	S	S	R	S	S	R	R	R	S	1	1.5	*blaZ, aacA-aphD, fusC, tet(K) sdrM*	hld, hl, hla, hlb, hIII	agrI; cap5; sak/scn; icaA/C/D
5222	**CC97-MRSA-V [fusC+]**	V	t267	-	02.05.16	Blood	R	R	R	S	S	S	S	S	S	R	R	R	S	2	3	*blaZ, aacA-aphD, fusC, sdrM*	hld, hl, hla, hlb, hIII	agrI; cap5; sak/scn; icaA/C/D
18455	**CC97-MRSA-V [fusC+]**	V	t267	-	19.12.16	Blood	R	R	R	S	S	S	R	S	S	R	S	R	S	1.5	1.5	*blaZ, vga(A), aacA-aphD, fusC, tet(K), sdrM*	hld, hl, hla, hlb, hIII	agrI; cap5; sak/scn; icaA/C/D
19775	**CC97-MRSA-V [fusC+]**	V	t267	-	11.06.17	Blood	R	R	R	S	S	S	S	S	S	R	S	R	S	1	1	*blaZ, aacA-aphD, fusC,sdrM*	hld, hl, hla, hlb, hIII	agrI; cap5; sak/scn; icaA/C/D
20287	**CC97-MRSA-V [fusC+]**	V	t267	-	27.08.17	Blood	R	R	R	S	S	S	S	S	S	R	S	R	S	1	1	*aacA-aphD, fusC,sdrM*	hld, hl, hla, hlb, hIII	agrI; cap5; sak/scn; icaA/C/D
20703	**CC97-MRSA-V [fusC+]**	V	t267	-	31.10.17	Blood	S	S	S	S	S	S	R	S	S	R	S	R	S	1	1	*aacA-aphD, fusC, tet(K), sdrM*	hld, hl, hla, hlb, hIII	agrI; cap5; sak/scn; icaA/C/D
20785	**CC97-MRSA-V [fusC+]**	V	t267	-	16.11.17	Blood	R	R	R	S	S	S	S	S	S	R	S	R	S	1.5	1.5	*aacA-aphD, fusC,sdrM*	hld, hl, hla, hlb, hIII	agrI; cap5; sak/scn; icaA/C/D
17748	**CC97-MRSA-V [fusC+]**	V	t2802	-	18.09.16	Blood	R	R	R	S	S	S	S	S	S	R	S	R	S	1.5	1.5	*blaZ, aacA-aphD, fusC, sdrM*	hld, hl, hla, hlb, hIII	agrI; cap5; sak/scn; icaA/C/D
17657	**CC97-MRSA-V [fusC+]**	V	t359	-	05.09.16	Blood	R	R	R	S	S	S	S	S	S	R	S	R	S	2	2	*blaZ, aacA-aphD, fusC, sdrM*	hld, hl, hla, hlb, hIII	agrI; cap5; sak/scn; icaA/C/D
18250	**CC97-MRSA-V [fusC+]**	V	t359	-	23.11.16	Blood	R	R	R	S	S	S	S	S	S	R	S	R	S	1	1	*aacA-aphD, fusC, sdrM*	hld, hl, hla, hlb, hIII	agrI; cap5; sak/scn; icaA/C/D
4916	**CC97-MRSA-V [fusC+]**	V	t267	-	04.04.16	Eye	R	R	R	R	I	S	S	S	S	R	S	R	S	1	2	*blaZ, erm(C), aacA-aphD, fusC, sdrM*	hld, hl, hla, hlb, hIII	agrI; cap5; sak/scn; icaA/C/D
17709	**CC97-MRSA-V [fusC+]**	V	t267	-	19.09.16	Eye	R	R	R	S	S	S	S	S	S	R	S	R	S	1	1.5	*blaZ, aacA-aphD, fusC, sdrM*	hld, hl, hla, hlb, hIII	agrI; cap5; sak/scn; icaA/C/D
19843	**CC97-MRSA-V [fusC+]**	V	t267	-	20.06.17	Eye	R	R	R	S	S	S	S	S	S	R	R	R	S	1	2	*blaZ, aacA-aphD, fusC,sdrM*	hld, hl, hla, hlb, hIII	agrI; cap5; sak/scn; icaA/C/D
5096	**CC97-MRSA-V [fusC+]**	V	t2734	-	21.04.16	Eye	R	R	R	S	S	S	S	S	S	R	S	R	S	1	1.5	*blaZ, aacA-aphD, fusC, sdrM*	hld, hl, hla, hlb, hIII	agrI; cap5; sak/scn; icaA/C/D
19755	**CC97-MRSA-V [fusC+]**	V	t701	-	06.06.17	Eye	R	R	R	S	S	S	S	S	S	R	S	R	S	2	2	*aacA-aphD, fusC,sdrM*	hld, hl, hla, hlb, hIII	agrI; cap5; sak/scn; icaA/C/D
19342	**CC97-MRSA-V [fusC+]**	V	ND	-	13.04.17	Fluid	R	R	R	S	S	S	R	S	S	R	S	R	S	1.5	3	*blaZ, aacA-aphD, fusC, tet(K), sdrM*	hld, hl, hla, hlb, hIII	agrI; cap5; sak/scn; icaA/C/D
4789	**CC97-MRSA-V [fusC+]**	V	t1814	-	21.3.2016	Groin	R	R	R	S	S	S	S	S	S	R	S	R	S	1.5	1.5	*blaZ, aacA-aphD, fusC, sdrM*	hld, hl, hla, hlb, hIII	agrI; cap5; sak/scn; icaA/C/D
17769	**CC97-MRSA-V [fusC+]**	V	t267	-	22.09.16	Groin	R	R	R	S	S	S	S	S	S	R	S	R	S	1.5	1.5	*blaZ, aacA-aphD, fusC, sdrM*	hld, hl, hla, hlb, hIII	agrI; cap5; sak/scn; icaA/C/D
18868	**CC97-MRSA-V [fusC+]**	V	t267	-	13.02.17	Groin	R	R	R	S	S	S	S	S	S	R	S	R	S	2	2	*blaZ, aacA-aphD, fusC,sdrM*	hld, hl, hla, hlb, hIII	agrI; cap5; sak/scn; icaA/C/D
18879	**CC97-MRSA-V [fusC+]**	V	t267	-	14.02.17	Groin	R	R	R	S	S	S	S	S	S	R	S	R	S	1.5	1.5	*blaZ, aacA-aphD, fusC,sdrM*	hld, hl, hla, hlb, hIII	agrI; cap5; sak/scn; icaA/C/D
19013	**CC97-MRSA-V [fusC+]**	V	t267	-	02.03.17	Groin	R	R	R	S	S	S	S	S	S	R	S	R	S	1.5	2	*blaZ, aacA-aphD, fusC,sdrM*	hld, hl, hla, hlb, hIII	agrI; cap5; sak/scn; icaA/C/D
18604	**CC97-MRSA-V [fusC+]**	V	t359	-	10.01.17	Groin	R	R	R	S	S	S	S	S	S	R	S	R	S	1	2	*blaZ, aacA-aphD, fusC, vga(A), sdrM*	hld, hl, hla, hlb, hIII	agrI; cap5; sak/scn; icaA/C/D
19302	**CC97-MRSA-V [fusC+]**	V	t359	-	04.04.17	Groin	R	R	R	S	S	S	R	S	S	R	S	R	S	2	1.5	*aacA-aphD, fusC, tet(K), sdrM*	hld, hl, hla, hlb, hIII	agrI; cap5; sak/scn; icaA/C/D
20424	**CC97-MRSA-V [fusC+]**	V	t521	-	18.09.17	Groin	R	R	R	S	S	S	R	S	S	R	S	R	S	1	1.5	*blaZ, aacA-aphD, fusC, tet(K,) sdrM*	hld, hl, hla, hlb, hIII	agrI; cap5; sak/scn; icaA/C/D
19366	**CC97-MRSA-V [fusC+]**	V	ND	-	16.04.17	HVS	R	R	R	S	S	S	R	S	S	R	S	R	S	2	2	*blaZ, aacA-aphD, fusC, tet(K), sdrM*	hld, hl, hla, hlb, hIII	agrI; cap5; sak/scn; icaA/C/D
18014	**CC97-MRSA-V [fusC+]**	V	t15069	-	27.10.16	HVS	R	S	S	S	S	S	S	S	S	R	S	R	S	1.5	1.5	*blaZ, fusC, sdrM*	hld, hl, hla, hlb, hIII	agrI; cap5; sak/scn; icaA/C/D
19818	**CC97-MRSA-V [fusC+]**	V	t17330	-	18.06.17	HVS	R	S	S	S	S	S	S	S	S	R	S	R	S	1.5	1.5	*blaZ, fusC, sdrM*	hld, hl, hla, hlb, hIII	agrI; cap5; sak/scn; icaA/C/D
5280	**CC97-MRSA-V [fusC+]**	V	t1965	-	11.05.16	HVS	S	R	R	S	S	S	S	S	S	R	R	R	S	1.5	2	*blaZ, aacA-aphD, fusC, sdrM*	hld, hl, hla, hlb, hIII	agrI; cap5; sak/scn; icaA/C/D
4584	**CC97-MRSA-V [fusC+]**	V	t267	-	28.02.16	HVS	R	R	R	S	S	S	S	S	S	R	S	R	S	2	3	*blaZ, aacA-aphD, fusC, sdrM*	hld, hl, hla, hlb, hIII	agrI; cap5; sak/scn; icaA/C/D
17911	**CC97-MRSA-V [fusC+]**	V	t267	-	16.10.16	HVS	R	R	R	S	S	S	S	S	S	R	S	R	S	1.5	2	*blaZ, aacA-aphD, fusC, sdrM*	hld, hl, hla, hlb, hIII	agrI; cap5; sak/scn; icaA/C/D
17272	**CC97-MRSA-V [fusC+]**	V	t267	-	14.1.16	HVS	R	R	R	R	S	S	R	S	S	R	S	R	S	1	1.5	*blaZ, msr(A), aacA-aphD, fusC, tet(K), sdrM*	hld, hl, hla, hlb, hIII	agrI; cap5; sak/scn; icaA/C/D
18326	**CC97-MRSA-V [fusC+]**	V	t267	-	04.12.16	HVS	R	R	R	S	S	S	S	S	S	R	S	R	S	1	1.5	*blaZ, aacA-aphD, fusC, sdrM*	hld, hl, hla, hlb, hIII	agrI; cap5; sak/scn; icaA/C/D
20610	**CC97-MRSA-V [fusC+]**	V	t267	-	15.10.17	HVS	R	R	R	S	S	S	S	S	S	R	S	R	S	1	2	*blaZ, aacA-aphD, fusC,sdrM*	hld, hl, hla, hlb, hIII	agrI; cap5; sak/scn; icaA/C/D
20562	**CC97-MRSA-V [fusC+]**	V	t359	-	09.10.17	HVS	R	R	R	S	S	S	S	S	S	R	S	R	S	1.5	1.5	*blaZ, aacA-aphD, fusC,sdrM*	hld, hl, hla, hlb, hIII	agrI; cap5; sak/scn; icaA/C/D
20626	**CC97-MRSA-V [fusC+]**	V	ND	-	22.10.17	Nasal	R	S	S	S	S	S	S	S	S	R	S	R	S	1.5	1.5	*blaZ, fusC,sdrM*	hld, hl, hla, hlb, hIII	agrI; cap5; sak/scn; icaA/C/D
17473	**CC97-MRSA-V [fusC+]**	V	t16486	-	11.08.16	Nasal	R	R	R	S	S	S	S	S	S	R	S	R	S	0.8	0.8	*blaZ, aacA-aphD, fusC, sdrM*	hld, hl, hla, hlb, hIII	agrI; cap5; sak/scn; icaA/C/D
18076	**CC97-MRSA-V [fusC+]**	V	t16606	-	01.11.16	Nasal	R	S	S	S	S	S	S	S	S	R	S	R	S	2	2	*blaZ, fusC, sdrM*	hld, hl, hla, hlb, hIII	agrI; cap5; sak/scn; icaA/C/D
19148	**CC97-MRSA-V [fusC+]**	V	t16606	-	19.03.17	Nasal	R	S	S	S	S	S	S	S	S	R	S	R	S	2	2	*blaZ, fusC, sdrM*	hld, hl, hla, hlb, hIII	agrI; cap5; sak/scn; icaA/C/D
18008	**CC97-MRSA-V [fusC+]**	V	t189	-	25.10.16	Nasal	R	R	R	S	S	S	S	S	S	R	S	R	S	2	2	*blaZ, aacA-aphD, fusC, sdrM*	hld, hl, hla, hlb, hIII	agrI; cap5; sak/scn; icaA/C/D
18010	**CC97-MRSA-V [fusC+]**	V	t2297	-	30.10.16	Nasal	R	S	S	S	S	S	S	S	S	R	S	R	S	2	2	*blaZ, fusC, sdrM*	hld, hl, hla, hlb, hIII	agrI; cap5; sak/scn; icaA/C/D
18558	**CC97-MRSA-V [fusC+]**	V	t2297	-	05.01.17	Nasal	R	S	S	S	S	S	S	S	S	R	S	R	S	1.5	2	*blaZ, fusC, sdrM*	hld, hl, hla, hlb, hIII	agrI; cap5; sak/scn; icaA/C/D
19622	**CC97-MRSA-V [fusC+]**	V	t2297	-	18.05.17	Nasal	R	S	S	S	S	S	S	S	S	R	S	R	S	2	2	*blaZ, fusC, sdrM*	hld, hl, hla, hlb, hIII	agrI; cap5; sak/scn; icaA/C/D
19479	**CC97-MRSA-V [fusC+]**	V	t2297	-	02.05.17	Nasal	R	S	S	S	S	S	S	S	S	R	S	R	S	1.5	2	*blaZ, fusC, sdrM*	hld, hl, hla, hlb, hIII	agrI; cap5; sak/scn; icaA/C/D
4563	**CC97-MRSA-V [fusC+]**	V	t267	-	24.2.16	Nasal	R	R	R	S	S	S	S	S	S	R	S	R	S	1.5	1.5	*blaZ, aacA-aphD, fusC, sdrM*	hld, hl, hla, hlb, hIII	agrI; cap5; sak/scn; icaA/C/D
4639	**CC97-MRSA-V [fusC+]**	V	t267	-	7.3.2016	Nasal	R	R	R	S	S	S	S	S	S	R	S	R	S	1.5	2	*blaZ, aacA-aphD, fusC, sdrM*	hld, hl, hla, hlb, hIII	agrI; cap5; sak/scn; icaA/C/D
17411	**CC97-MRSA-V [fusC+]**	V	t267	-	07.08.16	Nasal	R	R	R	S	S	S	S	S	S	R	S	R	LR	0.8	2	*blaZ, aacA-aphD, fusC, sdrM*	hld, hl, hla, hlb, hIII	agrI; cap5; sak/scn; icaA/C/D
18340	**CC97-MRSA-V [fusC+]**	V	t267	-	04.12.16	Nasal	R	R	R	S	S	S	S	S	S	R	S	R	S	2	2	*blaZ, aacA-aphD, fusC, sdrM*	hld, hl, hla, hlb, hIII	agrI; cap5; sak/scn; icaA/C/D
18435	**CC97-MRSA-V [fusC+]**	V	t267	-	18.12.16	Nasal	R	R	R	S	S	S	S	S	S	R	S	R	S	1.5	1	*blaZ, aacA-aphD, fusC, sdrM*	hld, hl, hla, hlb, hIII	agrI; cap5; sak/scn; icaA/C/D
18641	**CC97-MRSA-V [fusC+]**	V	t267	-	12.01.17	Nasal	R	R	R	S	S	S	S	S	S	R	S	R	S	1.5	1.5	*blaZ, aacA-aphD, fusC, sdrM*	hld, hl, hla, hlb, hIII	agrI; cap5; sak/scn; icaA/C/D
18908	**CC97-MRSA-V [fusC+]**	V	t267	-	19.02.17	Nasal	R	S	S	S	S	S	S	S	S	R	S	R	S	1.5	1	*blaZ, fusC, sdrM*	sek, seq; hld, hl, hla, hlb, hIII	agrI; cap5; sak/scn; icaA/C/D
18846	**CC97-MRSA-V [fusC+]**	V	t267	-	12.02.17	Nasal	R	R	R	S	S	S	R	S	S	R	S	R	S	1.5	2	*aacA-aphD, fusC, tet(K), sdrM*	hld, hl, hla, hlb, hIII	agrI; cap5; sak/scn; icaA/C/D
18796	**CC97-MRSA-V [fusC+]**	V	t267	-	02.02.17	Nasal	R	R	R	S	S	S	S	S	S	R	S	R	S	1.5	1.5	*blaZ, aacA-aphD, fusC,sdrM*	hld, hl, hla, hlb, hIII	agrI; cap5; sak/scn; icaA/C/D
19482	**CC97-MRSA-V [fusC+]**	V	t267	-	02.05.17	Nasal	R	R	R	S	S	S	S	S	S	R	S	R	S	2	2	*blaZ, aacA-aphD, fusC,sdrM*	hld, hl, hla, hlb, hIII	agrI; cap5; sak/scn; icaA/C/D
20113	**CC97-MRSA-V [fusC+]**	V	t267	-	01.08.17	Nasal	R	R	R	S	S	S	S	S	S	R	S	R	S	1	0.5	*blaZ, aacA-aphD, fusC,sdrM*	hld, hl, hla, hlb, hIII	agrI; cap5; sak/scn; icaA/C/D
20535	**CC97-MRSA-V [fusC+]**	V	t267	-	03.10.17	Nasal	R	R	R	S	S	S	S	S	S	R	S	R	S	1.5	1.5	*blaZ, aacA-aphD, fusC,sdrM*	hld, hl, hla, hlb, hIII	agrI; cap5; sak/scn; icaA/C/D
20817	**CC97-MRSA-V [fusC+]**	V	t267	-	16.11.17	Nasal	R	R	R	S	S	S	S	S	S	R	S	R	S	2	1	*blaZ, aacA-aphD, fusC,sdrM*	hld, hl, hla, hlb, hIII	agrI; cap5; sak/scn; icaA/C/D
20762	**CC97-MRSA-V [fusC+]**	V	t267	-	12.11.17	Nasal	R	R	R	S	S	S	S	S	S	S	S	R	S	1.5	1.5	*aacA-aphD, fusC,sdrM*	hld, hl, hla, hlb, hIII	agrI; cap5; sak/scn; icaA/C/D

20764	**CC97-MRSA-V [fusC+]**	V	t267	-	12.11.17	Nasal	R	R	R	S	S	S	S	S	S	R	S	R	S	1	1.5	*fusC,sdrM*	hld, hl, hla, hlb, hIII	agrI; cap5; sak/scn; icaA/C/D
20765	**CC97-MRSA-V [fusC+]**	V	t267	-	12.11.17	Nasal	R	R	R	S	S	S	S	S	S	R	S	R	S	1	1.5	*aacA-aphD, fusC,sdrM*	hld, hl, hla, hlb, hIII	agrI; cap5; sak/scn; icaA/C/D
20751	**CC97-MRSA-V [fusC+]**	V	t267	-	09.11.17	Nasal	R	S	S	S	S	S	R	S	S	R	S	R	S	1	1	*aacA-aphD, fusC, tet(K), sdrM*	hld, hl, hla, hlb, hIII	agrI; cap5; sak/scn; icaA/C/D
4689	**CC97-MRSA-V [fusC+]**	V	t359	-	10.3.2016	Nasal	R	R	R	S	S	S	R	S	S	R	S	R	S	1.5	1.5	*blaZ, aacA-aphD, fusC, tet(K), sdrM*	hld, hl, hla, hlb, hIII	agrI; cap5; sak/scn; icaA/C/D
5515	**CC97-MRSA-V [fusC+]**	V	t359	-	30.05.16	Nasal	R	R	R	S	S	S	R	S	S	R	S	R	S	1.5	1	*blaZ, aacA-aphD, fusC, tet(K), sdrM*	hld, hl, hla, hlb, hIII	agrI; cap5; sak/scn; icaA/C/D
18114	**CC97-MRSA-V [fusC+]**	V	t359	-	08.11.16	Nasal	R	R	R	S	S	S	S	S	S	R	S	R	S	1.5	2	*blaZ, aacA-aphD, fusC, sdrM*	hld, hl, hla, hlb, hIII	agrI; cap5; sak/scn; icaA/C/D
20136	**CC97-MRSA-V [fusC+]**	V	t359	-	03.08.17	Nasal	R	S	S	S	S	S	S	S	S	R	S	R	S	1.5	3	*blaZ, fusC,sdrM*	hld, hl, hla, hlb, hIII	agrI; cap5; sak/scn; icaA/C/D
4688	**CC97-MRSA-V [fusC+]**	V	t527	-	13.3.2016	Nasal	R	R	R	S	S	S	S	S	S	R	S	R	S	1.5	1	*blaZ, aacA-aphD, fusC, sdrM*	hld, hl, hlb, hIII	agrI; cap5; sak/scn; icaA/C/D
17822	**CC97-MRSA-V [fusC+]**	V	t1814	-	03.10.16	Others	R	R	R	S	S	S	S	S	S	R	S	R	S	2	1.5	*blaZ, vga(A), aacA-aphD, fusC, sdrM*	hld, hl, hla, hlb, hIII	agrI; cap5; sak/scn; icaA/C/D
17398	**CC97-MRSA-V [fusC+]**	V	t189	-	04.08.16	Others	R	R	R	R	C	R	S	S	S	R	S	R	S	1	1.5	*blaZ, erm(C), aacA-aphD, fusC, cat, sdrM*	hld, hl, hla, hlb, hIII	agrI; cap5; sak/scn; icaA/C/D
17417	**CC97-MRSA-V [fusC+]**	V	t267	-	07.08.16	Others	R	R	R	S	S	S	S	S	S	R	S	R	S	1.5	2	*blaZ, aacA-aphD, fusC, sdrM*	hld, hl, hla, hlb, hIII	agrI; cap5; sak/scn; icaA/C/D
19039	**CC97-MRSA-V [fusC+]**	V	t267	-	07.03.17	Others	R	R	R	S	S	S	S	S	S	R	S	R	S	1.5	1.5	*blaZ, aacA-aphD, fusC,sdrM*	hld, hl, hla, hlb, hIII	agrI; cap5; sak/scn; icaA/C/D
19609	**CC97-MRSA-V [fusC+]**	V	t267	-	18.05.17	Others	R	R	R	S	S	S	S	S	S	R	S	R	S	2	2	*blaZ, aacA-aphD, fusC,sdrM*	hld, hl, hla, hlb, hIII	agrI; cap5; sak/scn; icaA/C/D
18304	**CC97-MRSA-V [fusC+]**	V	t2770	-	30.11.16	Others	R	S	S	S	S	S	S	S	S	R	S	R	S	0.5	0.8	*blaZ, fusC, sdrM*	hld, hl, hla, hlb, hIII	agrI; cap5; sak/scn; icaA/C/D
20844	**CC97-MRSA-V [fusC+]**	V	t359	-	23.11.17	Others	R	R	R	S	S	S	S	S	S	R	S	R	S	1.5	2	*aacA-aphD, fusC,sdrM*	hld, hl, hla, hlb, hIII	agrI; cap5; sak/scn; icaA/C/D
18070	**CC97-MRSA-V [fusC+]**	V	t9638	-	01.11.16	Others	R	R	R	S	S	S	R	S	S	R	R	R	S	1.5	2	*blaZ, aacA-aphD, fusC, tet(K), sdrM*	hld, hl, hla, hlb, hIII	agrI; cap5; sak/scn; icaA/C/D
20729	**CC97-MRSA-V [fusC+]**	V	t189	-	05.11.17	Pus	R	R	R	S	S	S	S	S	S	R	S	R	S	1	1.5	*blaZ, aacA-aphD, fusC,sdrM*	selo, egc; hld, hl, hla, hlb, hIII	agrI; cap5; sak/scn; icaA/C/D
17830	**CC97-MRSA-V [fusC+]**	V	t2297	-	04.10.16	Pus	R	S	S	S	S	S	S	S	S	R	S	R	S	2	2	*blaZ, fusC, sdrM*	hld, hl, hla, hlb, hIII	agrI; cap5; sak/scn; icaA/C/D
18783	**CC97-MRSA-V [fusC+]**	V	t2297	-	02.02.17	Pus	R	S	S	S	S	S	S	S	S	R	S	R	S	1	2	*fusC, sdrM*	hld, hl, hla, hlb, hIII	agrI; cap5; sak/scn; icaA/C/D
19779	**CC97-MRSA-V [fusC+]**	V	t2297	-	11.06.17	Pus	R	S	S	S	S	S	S	S	S	R	S	R	S	1.5	2	*fusC, sdrM*	hld, hl, hla, hlb, hIII	agrI; cap5; sak/scn; icaA/C/D
18184	**CC97-MRSA-V [fusC+]**	V	t267	-	20.11.16	Pus	R	R	R	S	S	S	S	S	S	R	S	R	S	2	2	*blaZ, aacA-aphD, fusC, sdrM*	hld, hl, hla, hlb, hIII	agrI; cap5; sak/scn; icaA/C/D
18571	**CC97-MRSA-V [fusC+]**	V	t359	-	02.01.17	Pus	R	R	R	S	S	S	R	S	S	S	R	R	S	1.5	1.5	*aacA-aphD, fusC, tet(K), sdrM*	hld, hl, hla, hlb, hIII	agrI; cap5; sak/scn; icaA/C/D
18785	**CC97-MRSA-V [fusC+]**	V	t359	-	01.02.17	Pus	R	R	R	S	S	S	S	S	S	R	S	R	S	1	1	*blaZ, aacA-aphD, fusC,sdrM*	hld, hl, hla, hlb, hIII	agrI; cap5; sak/scn; icaA/C/D
19511	**CC97-MRSA-V [fusC+]**	V	t359	-	07.05.17	Pus	R	R	R	S	S	S	S	S	S	R	S	R	S	2	2	*blaZ, aacA-aphD, fusC,sdrM*	hld, hl, hla, hlb, hIII	agrI; cap5; sak/scn; icaA/C/D
19669	**CC97-MRSA-V [fusC+]**	V	t17281	-	22.05.17	Skin	R	R	R	S	S	S	S	S	S	R	S	R	S	2	2	*blaZ, aacA-aphD, fusC,sdrM*	hld, hl, hla, hlb, hIII	agrI; cap5; sak/scn; icaA/C/D
4671	**CC97-MRSA-V [fusC+]**	V	t267	-	8.3.2016	Skin	S	R	R	R	C	S	S	S	S	R	S	R	S	1.5	1	*aacA-aphD, fusC, sdrM*	hld, hl, hla, hlb, hIII	agrI; cap5; sak/scn; icaA/C/D
19201	**CC97-MRSA-V [fusC+]**	V	t267	-	26.03.17	Skin	S	R	R	S	S	S	S	S	S	R	S	R	S	1.5	1.5	*aacA-aphD, fusC,sdrM*	hld, hl, hla, hlb, hIII	agrI; cap5; sak/scn; icaA/C/D
17580	**CC97-MRSA-V [fusC+]**	V	t359	-	25.08.16	Skin	R	R	R	S	S	S	S	S	S	R	S	R	S	1.5	1	*blaZ, aacA-aphD, fusC, sdrM*	hld, hl, hla, hlb, hIII	agrI; cap5; sak/scn; icaA/C/D
17675	**CC97-MRSA-V [fusC+]**	V	t359	-	07.09.16	Skin	R	R	R	S	S	S	S	S	S	R	S	R	S	1.5	1.5	*blaZ, aacA-aphD, fusC, sdrM*	hld, hl, hla, hlb, hIII	agrI; cap5; sak/scn; icaA/C/D
18802	**CC97-MRSA-V [fusC+]**	V	t267	-	07.02.17	Sputum	R	R	R	S	S	S	S	S	S	R	S	R	S	2	2	*blaZ, aacA-aphD, fusC,sdrM*	hld, hl, hla, hlb, hIII	agrI; cap5; sak/scn; icaA/C/D
20619	**CC97-MRSA-V [fusC+]**	V	t267	-	17.10.17	Sputum	R	S	S	S	S	S	S	S	S	R	S	R	S	1	1.5	*blaZ, fusC,sdrM*	hld, hl, hla, hlb, hIII	agrI; cap5; sak/scn; icaA/C/D
4613	**CC97-MRSA-V [fusC+]**	V	t359	-	2.3.2016	Sputum	R	R	R	S	S	S	S	S	S	R	S	R	S	1	1.5	*blaZ, aacA-aphD, fusC, fusB, sdrM*	tst1; hld, hl, hla, hlb, hIII	agrI; cap5; sak/scn; icaA/C/D
5512	**CC97-MRSA-V [fusC+]**	V	t359	-	30.05.16	Sputum	R	R	R	R	S	S	S	S	S	R	S	R	S	1.5	1.5	*blaZ, aacA-aphD, fusC, sdrM*	hld, hl, hla, hlb, hIII	agrI; cap5; sak/scn; icaA/C/D
5612	**CC97-MRSA-V [fusC+]**	V	t359	-	09.06.16	Sputum	R	R	R	S	S	S	S	S	S	R	S	R	S	1	1.5	*blaZ, aacA-aphD, fusC, sdrM*	hld, hl, hla, hlb, hIII	agrI; cap5; sak/scn; icaA/C/D
17916	**CC97-MRSA-V [fusC+]**	V	t359	-	16.10.16	Sputum	R	R	R	S	S	S	S	S	S	R	S	R	S	2	2	*blaZ, aacA-aphD, fusC, sdrM*	hld, hl, hla, hlb, hIII	agrI; cap5; sak/scn; icaA/C/D
18839	**CC97-MRSA-V [fusC+]**	V	t359	-	05.02.17	Sputum	R	R	R	S	S	S	S	S	S	R	S	R	S	1.5	1.5	*blaZ, aacA-aphD, fusC,sdrM*	hld, hl, hla, hlb, hIII	agrI; cap5; sak/scn; icaA/C/D
19472	**CC97-MRSA-V [fusC+]**	V	t359	-	26.04.17	Sputum	R	R	R	S	S	S	S	S	S	R	S	R	S	2	1.5	*blaZ, aacA-aphD, fusC,sdrM*	hld, hl, hla, hlb, hIII	agrI; cap5; sak/scn; icaA/C/D
19643	**CC97-MRSA-V [fusC+]**	V	t359	-	21.05.17	Sputum	R	R	R	S	S	S	S	S	S	R	S	R	S	2	2	*blaZ, aacA-aphD, fusC,sdrM*	hld, hl, hla, hlb, hIII	agrI; cap5; sak/scn; icaA/C/D
20723	**CC97-MRSA-V [fusC+]**	V	t359	-	05.11.17	Sputum	R	R	R	S	S	S	S	S	S	R	S	R	S	1.5	1.5	*blaZ, aacA-aphD, fusC,sdrM*	hld, hl, hla, hlb, hIII	agrI; cap5; sak/scn; icaA/C/D
5165	**CC97-MRSA-V [fusC+]**	V	t521	-	28.04.16	Sputum	R	R	R	S	S	S	S	S	S	R	S	R	S	1	1.5	*blaZ, aacA-aphD, fusC, cat, vanB, sdrM*	sed, hld, hl, hla, hlb, hIII	agrI; cap5; sak/scn; icaA/C/D; arcD-S
19526	**CC97-MRSA-V [fusC+]**	V	t17282	-	07.05.17	Swab	R	S	S	S	S	S	R	S	S	R	S	R	S	2	3	*blaZ, tet(K), fusC, sdrM*	sek, seq; hld, hl, hla, hlb, hIII	agrI; cap5; sak/scn; icaA/C/D
18182	**CC97-MRSA-V [fusC+]**	V	t267	-	17.11.16	Swab	R	R	R	S	S	S	S	S	S	R	R	R	S	1	1	*blaZ, aacA-aphD, fusC, sdrM*	hld, hl, hla, hlb, hIII	agrI; cap5; sak/scn; icaA/C/D
5535	**CC97-MRSA-V [fusC+]**	V	t267	-	01.06.16	Throat	R	R	R	S	S	S	R	S	S	R	R	R	S	1	1.5	*blaZ, aacA-aphD, fusC, tet(K), sdrM*	sed, hld, hl, hla, hlb, hIII	agrI; cap5; sak/scn; icaA/C/D
18499	**CC97-MRSA-V [fusC+]**	V	t267	-	20.12.16	Throat	S	R	R	S	S	S	R	S	S	R	S	R	S	0.8	1.5	*aacA-aphD, fusC, tet(K), sdrM*	hld, hl, hla, hlb, hIII	agrI; cap5; sak/scn; icaA/C/D
18901	**CC97-MRSA-V [fusC+]**	V	t267	-	19.02.17	Throat	R	R	R	S	S	S	S	S	S	S	S	R	S	1.5	1.5	*blaZ, aacA-aphD, fusC,sdrM*	hld, hl, hla, hlb, hIII	agrI; cap5; sak/scn; icaA/C/D
18845	**CC97-MRSA-V [fusC+]**	V	t267	-	09.02.17	Throat	S	R	R	S	S	S	R	S	S	R	S	R	S	1	1.5	*aacA-aphD, fusC, tet(K), sdrM*	hld, hl, hla, hlb, hIII	agrI; cap5; sak/scn; icaA/C/D
18676	**CC97-MRSA-V [fusC+]**	V	t359	-	18.01.17	Throat	S	R	R	S	S	S	R	S	S	R	S	R	S	1	1.5	*aacA-aphD, fusC, tet(K), sdrM*	hld, hl, hla, hlb, hIII	agrI; cap5; sak/scn; icaA/C/D
5212	**CC97-MRSA-V [fusC+]**	V	t376	-	02.05.16	Throat	R	R	R	S	S	S	S	S	S	R	S	R	S	1.5	2	*blaZ, aacA-aphD, fusC, sdrM*	hld, hl, hla, hlb, hIII	agrI; cap5; sak/scn; icaA/C/D
5279	**CC97-MRSA-V [fusC+]**	V	t376	-	11.05.16	Throat	R	R	R	S	S	S	S	S	S	R	S	R	S	2	1.5	*blaZ, aacA-aphD, fusC, sdrM*	hld, hl, hla, hlb, hIII	agrI; cap5; sak/scn; icaA/C/D
17796	**CC97-MRSA-V [fusC+]**	V	t267	-	27.09.16	Tracheal	R	R	R	S	S	S	R	S	S	R	S	R	S	1.5	1.5	*blaZ, aacA-aphD, fusC, tet(K), sdrM*	hld, hl, hla, hlb, hIII	agrI; cap5; sak/scn; icaA/C/D
17665	**CC97-MRSA-V [fusC+]**	V	t267	-	05.09.16	Tracheal	R	S	S	S	S	S	S	S	S	R	S	R	LR	2	2	*blaZ, aacA-aphD, fusC, sdrM*	hld, hl, hla, hlb, hIII	agrI; cap5; sak/scn; icaA/C/D
17771	**CC97-MRSA-V [fusC+]**	V	t359	-	22.09.16	Tracheal	R	R	R	S	S	S	S	S	S	R	S	R	S	1	1.5	*aacA-aphD, fusC, sdrM*	hld, hl, hla, hlb, hIII	agrI; cap5; sak/scn; icaA/C/D
19357	**CC97-MRSA-V [fusC+]**	V	ND	-	13.04.17	Unknown	R	S	S	S	S	S	S	S	S	R	R	R	S	2	2	*fusC, sdrM*	hld, hl, hla, hlb, hIII	agrI; cap5; sak/scn; icaA/C/D
19002	**CC97-MRSA-V [fusC+]**	V	t189	-	02.03.17	Unknown	R	R	R	S	S	S	S	S	S	R	S	R	S	2	1.5	*blaZ, aacA-aphD, fusC,sdrM*	hld, hl, hla, hlb, hIII	agrI; cap5; sak/scn; icaA/C/D
20823	**CC97-MRSA-V [fusC+]**	V	t2297	-	20.11.17	Unknown	R	S	S	S	S	S	S	S	S	R	S	R	S	1.5	1.5	*fusC, sdrM*	hld, hl, hla, hlb, hIII	agrI; cap5; sak/scn; icaA/C/D
17876	**CC97-MRSA-V [fusC+]**	V	t267	-	11.10.16	Unknown	R	S	S	S	S	S	S	S	S	S	S	R	S	1.5	2	*blaZ, aacA-aphD, fusC, sdrM*	hld, hl, hla, hlb, hIII	agrI; cap5; sak/scn; icaA/C/D
18469	**CC97-MRSA-V [fusC+]**	V	t267	-	20.12.16	Unknown	R	S	S	S	S	S	S	S	S	R	S	R	S	1.5	1.5	*blaZ, aacA-aphD, fusC, sdrM*	hld, hl, hla, hlb, hIII	agrI; cap5; sak/scn; icaA/C/D
19241	**CC97-MRSA-V [fusC+]**	V	t267	-	28.03.17	Unknown	R	R	R	S	S	S	S	S	S	R	S	R	S	1.5	1.5	*blaZ, aacA-aphD, fusC,sdrM*	hld, hl, hla, hlb, hIII	agrI; cap5; sak/scn; icaA/C/D
19408	**CC97-MRSA-V [fusC+]**	V	t267	-	20.04.17	Unknown	R	S	S	S	S	S	S	S	S	R	S	R	S	2	2	*blaZ, fusC,sdrM*	hld, hl, hla, hlb, hIII	agrI; cap5; sak/scn; icaA/C/D
5157	**CC97-MRSA-V [fusC+]**	V	t359	-	28.04.16	Unknown	R	R	R	S	S	S	R	S	S	R	S	R	S	0.8	2	*blaZ, aacA-aphD, fusC, tet(K), sdrM*	hld, hl, hla, hlb, hIII	agrI; cap5; sak/scn; icaA/C/D
18627	**CC97-MRSA-V [fusC+]**	V	t267	-	11.01.17	Urine	R	R	R	S	S	S	R	S	S	R	S	R	S	1	0.8	*blaZ, aacA-aphD, fusC, tet(K), sdrM*	hld, hl, hla, hlb, hIII	agrI; cap5; sak/scn; icaA/C/D
20378	**CC97-MRSA-V [fusC+]**	V	t521	-	12.09.17	Urine	R	R	R	S	S	S	R	S	S	R	S	R	S	1.5	1	*blaZ, aacA-aphD, fusC, tet(K), sdrM*	hld, hl, hla, hlb, hIII	agrI; cap5; sak/scn; icaA/C/D
20370	**CC97-MRSA-V [fusC+]**	V	ND	-	12.09.17	Wound	R	R	R	S	S	S	S	S	S	R	S	R	S	0.8	1.5	*blaZ, aacA-aphD, fusC,sdrM*	hld, hl, hla, hlb, hIII	agrI; cap5; sak/scn; icaA/C/D
17839	**CC97-MRSA-V [fusC+]**	V	t044	-	05.10.16	Wound	S	R	R	S	S	S	S	S	S	R	S	R	S	1.5	1	*aacA-aphD, fusC, sdrM*	hld, hl, hla, hlb, hIII	agrI; cap5; sak/scn; icaA/C/D
4665	**CC97-MRSA-V [fusC+]**	V	t267	-	8.3.2016	Wound	R	R	R	S	S	S	R	S	S	R	S	R	S	1.5	1	*blaZ, aacA-aphD, fusC, tet(K), sdrM*	hld, hl, hla, hlb, hIII	agrI; cap5; sak/scn; icaA/C/D
17484	**CC97-MRSA-V [fusC+]**	V	t267	-	15.08.16	Wound	R	R	R	S	S	S	S	S	S	R	S	R	S	1.5	1	*blaZ, aacA-aphD, fusC, sdrM*	hld, hl, hlb, hIII	agrI; cap5; sak/scn; icaA/C/D

17470	**CC97-MRSA-V [fusC+]**	V	t267	-	11.08.16	Wound	R	R	R	S	S	S	R	S	S	R	S	R	S	2	2	*blaZ, aacA-aphD, fusC, tet(K), sdrM*	hld, hl, hla, hlb, hIII	agrI; cap5; sak/scn; icaA/C/D
17636	**CC97-MRSA-V [fusC+]**	V	t267	-	01.09.16	Wound	R	R	R	S	S	S	R	S	S	R	S	R	S	1.5	1.5	*blaZ, aacA-aphD, fusC, tet(K), sdrM*	hld, hl, hla, hlb, hIII	agrI; cap5; sak/scn; icaA/C/D
17970	**CC97-MRSA-V [fusC+]**	V	t267	-	19.10.16	Wound	R	R	R	S	S	S	S	S	S	R	S	R	S	1.5	1	*blaZ, aacA-aphD, fusC, sdrM*	hld, hl, hla, hlb, hIII	agrI; cap5; sak/scn; icaA/C/D
17979	**CC97-MRSA-V [fusC+]**	V	t267	-	23.10.16	Wound	R	R	R	S	S	S	S	S	S	R	S	R	S	2	2	*blaZ, aacA-aphD, fusC, sdrM*	hld, hl, hla, hlb, hIII	agrI; cap5; sak/scn; icaA/C/D
18548	**CC97-MRSA-V [fusC+]**	V	t267	-	02.01.17	Wound	R	R	R	S	S	S	S	S	S	R	S	R	S	2	2	*blaZ, aacA-aphD, fusC, sdrM*	hld, hl, hla, hlb, hIII	agrI; cap5; sak/scn; icaA/C/D
19604	**CC97-MRSA-V [fusC+]**	V	t267	-	21.05.17	Wound	R	R	R	S	S	S	S	S	S	R	S	R	S	1.5	2	*blaZ, aacA-aphD, fusC,sdrM*	hld, hl, hla, hlb, hIII	agrI; cap5; sak/scn; icaA/C/D
19793	**CC97-MRSA-V [fusC+]**	V	t267	-	13.06.17	Wound	R	R	R	R	C	S	S	S	S	R	S	R	S	1.5	2	*blaZ, erm(C), aacA-aphD, fusC,sdrM*	hld, hl, hla, hlb, hIII	agrI; cap5; sak/scn; icaA/C/D
20774	**CC97-MRSA-V [fusC+]**	V	t267	-	14.11.17	Wound	R	R	R	S	S	S	S	S	S	R	S	R	S	1.5	1.5	*aacA-aphD, fusC,sdrM*	hld, hl, hla, hlb, hIII	agrI; cap5; sak/scn; icaA/C/D
19350	**CC97-MRSA-V [fusC+]**	V	t359	-	13.04.17	Wound	R	R	R	S	S	S	S	S	S	R	R	R	S	1.5	1.5	*blaZ, aacA-aphD, fusC,sdrM*	hld, hl, hla, hlb, hIII	agrI; cap5; sak/scn; icaA/C/D
19868	**CC97-MRSA-V [fusC+]**	V	t359	-	29.06.17	Wound	R	R	R	S	S	S	S	S	S	R	S	R	S	1	1.5	*blaZ, aacA-aphD, fusC,sdrM*	hld, hl, hla, hlb, hIII	agrI; cap5; sak/scn; icaA/C/D
20680	**CC97-MRSA-V [fusC+]**	V	t359	-	30.10.17	Wound	R	R	R	S	S	S	S	S	S	R	S	R	S	1.5	2	*blaZ, aacA-aphD, fusC, fosB, sdrM*	hld, hl, hla, hlb, hIII	agrI; cap5; sak/scn; icaA/C/D
20548	**CC97-MRSA-V [fusC+]**	V	t359	-	05.10.17	Wound	R	R	R	S	S	S	S	S	S	R	S	R	S	1.5	1.5	*blaZ, aacA-aphD, fusC,sdrM*	hld, hl, hla, hlb, hIII	agrI; cap5; sak/scn; icaA/C/D
20709	**CC97-MRSA-V [fusC+]**	V	t521	-	02.11.17	Wound	R	R	R	S	S	S	S	S	S	R	S	R	S	1.5	1.5	*blaZ, aacA-aphD, fusC,sdrM*	hld, hl, hla, hlb, hIII	agrI; cap5; sak/scn; icaA/C/D
18488	**CC97-MSSA**	V	t16903	-	22.12.06	HVS	R	R	R	R	C	S	R	S	S	S	S	R	LR	1.5	1.5	*blaZ, fosB, sdrM*	sea (N315); hld, hl, hla, hlb, hIII	agrI; cap5; sak/scn; icaA/C/D

cc
